# Predictors of poor retention on antiretroviral therapy as a major HIV drug resistance early warning indicator in Cameroon: results from a nationwide systematic random sampling

**DOI:** 10.1186/s12879-016-1991-3

**Published:** 2016-11-15

**Authors:** Serge Clotaire Billong, Joseph Fokam, Calixte Ida Penda, Salmon Amadou, David Same Kob, Edson-Joan Billong, Vittorio Colizzi, Alexis Ndjolo, Anne-Cecile Zoung-Kani Bisseck, Jean-Bosco Nfetam Elat

**Affiliations:** 1 National HIV drug resistance surveillance and prevention Working Group (HIVDR-WG), Ministry of Public Health, Yaoundé, Cameroon; 2 Faculty of Medicine and Biomedical Sciences (FMBS), University of Yaoundé 1, Yaoundé, Cameroon; 3 National AIDS Control Committee, Ministry of Public Health, Yaoundé, Cameroon; 4 Chantal BIYA International Reference Centre (CIRCB) for research on HIV/AIDS prevention and management, Ministry of Public Health, Yaoundé, Cameroon; 5 Department of Experimental Medicine and Surgery, Faculty of Medicine and Surgery, University of Rome Tor Vergata, Rome, Italy; 6 Faculty of Medicine and Pharmaceutical sciences (FMSP), University of Douala, Douala, Cameroon; 7 Laquintinie Hospital of Douala, Douala, Cameroon; 8 Faculty of Medicine, University of Antananarivo, Antananarivo, Madagascar; 9 UNESCO Biotechnology Multidisciplinary Board, and Department of Biology and Pathology, Faculty of Sciences, University of Rome Tor Vergata, Rome, Italy; 10 Division of Operational Health Research, Ministry of Public Health, Yaounde, Cameroon

**Keywords:** Retention in care, Antiretroviral therapy, HIV drug resistance early warning indicator, Cameroon, Random sampling

## Abstract

**Background:**

Retention on lifelong antiretroviral therapy (ART) is essential in sustaining treatment success while preventing HIV drug resistance (HIVDR), especially in resource-limited settings (RLS). In an era of rising numbers of patients on ART, mastering patients in care is becoming more strategic for programmatic interventions. Due to lapses and uncertainty with the current WHO sampling approach in Cameroon, we thus aimed to ascertain the national performance of, and determinants in, retention on ART at 12 months.

**Methods:**

Using a systematic random sampling, a survey was conducted in the ten regions (56 sites) of Cameroon, within the “*reporting period*” of October 2013–November 2014, enrolling 5005 eligible adults and children. Performance in retention on ART at 12 months was interpreted following the definition of HIVDR early warning indicator: excellent (>85%), fair (85–75%), poor (<75); and factors with *p*-value < 0.01 were considered statistically significant.

**Results:**

Majority (74.4%) of patients were in urban settings, and 50.9% were managed in reference treatment centres. Nationwide, retention on ART at 12 months was 60.4% (2023/3349); only six sites and one region achieved acceptable performances. Retention performance varied in reference treatment centres (54.2%) vs. management units (66.8%), *p* < 0.0001; male (57.1%) vs. women (62.0%), *p* = 0.007; and with WHO clinical stage I (63.3%) vs. other stages (55.6%), *p* = 0.007; but neither for age (adults [60.3%] vs. children [58.8%], *p* = 0.730) nor for immune status (CD4_351–500_ [65.9%] vs. other CD4-staging [59.86%], *p* = 0.077).

**Conclusions:**

Poor retention in care, within 12 months of ART initiation, urges active search for lost-to-follow-up targeting preferentially male and symptomatic patients, especially within reference ART clinics. Such sampling strategy could be further strengthened for informed ART monitoring and HIVDR prevention perspectives.

**Electronic supplementary material:**

The online version of this article (doi:10.1186/s12879-016-1991-3) contains supplementary material, which is available to authorized users.

## Background

With a total of 145,038 adults and children receiving antiretroviral therapy (ART) out of 623,350 people living with HIV (PLHIV) in Cameroon, scalability of ART is a burning priority to the national AIDS programme [1, 2]. Of note, Cameroon recently adopted the 2013 World Health Organization (WHO) public health recommendations of the consolidated guidelines on the use of antiretroviral drugs for treating and preventing HIV infection, increasing eligibility of ART based on CD4 count (from the former threshold of ≤350 to ≤500 cells/mm^3^) and providing universal access to lifelong ART for HIV-infected children under five years and for HIV-infected pregnant women in the context of the prevention of mother-to-child transmission (PMTCT) of HIV (transitioning from Option-A to Option-B+) [3, 4]. These implications would lead to ~80% enrolment on ART among people diagnosed HIV positive, thus suggesting the need of innovative strategies for effective and successful programmatic ART uptakes [3, 5].

Ensuring a successful ART programme in this new therapeutic era requires evidence-based findings on programmatic, clinic and patient factors associated with national performance [5, 6]. Our experience in assessing these factors locally, using early warning indicators (EWI) of HIV drug resistance (HIVDR), consistently reveals pharmacy stock-outs and delayed pill pick-up as major factors favoring HIVDR emergence; while prescribing/dispensing practices remain appropriate, indicators of retention in care and lost to follow-up are fluctuating, making it challenging to design and implement reasonable public health interventions nationwide [7–9], especially with the changing paradigms in ART [5].

Retaining patients on lifelong ART, is an essential component in HIVDR prevention through adherence promotion, considerably improved the clinical and immunological outcomes [10, 11], reduced lost to follow-up and prolong the life expectancy of treated-patients [3]. As this indicator remains with uncertainty [7–9], it became crucial for the national AIDS programme to delineate the country performance in patient retention on ART as well as related determinants. Such investigation would provide a reliable representativeness of Cameroon’s capacity in retaining patients on ART and prompt real time corrective measures while transitioning to current treatment strategies [3, 4].

In a context of declining trends in HIVDR EWI, the number of HIV clinics with satisfactory performance in patient retention on ART dropped drastically (from 70 to 0%) in selected sites of our national ART programme [8]. Moreover, retention on ART varied significantly between urban and rural settings in Cameroon [9], thereby stressing the necessity of in-depth understanding of disparities surrounding this key indicator of patient adherence to ART program [5, 6].

In terms of programmatic perspectives, an accurate mastering of patients in care provide a better accountability and planning for ARV drug procurement, thereby reducing events of discontinuous drug supply as previously reported [7–9]. Of note, drug shortage suggests ART interruption, which is a direct indicator of suboptimal ARV adherence and higher risks of HIVDR emergence in a context where low-genetic barrier drugs (i.e. lamivudine [3TC], emtricitabine [FTC], nevirapine [NVP], efavirenz [EFV]) are widely used for patient management [3, 4]. Therefore, poor retention on ART automatically results in higher lost to follow-up and HIVDR emergence and subsequent transmission to newly infected people within the communities [4, 5].

To minimize lost to follow-up and its associated risks of HIVDR, the WHO recommends to evaluate “*retention in care at 12 months of ART*” as part of five simplified EWIs (*on- time pill pick-up*; *retention in care at 12 months*; *pharmacy stock-outs; dispensing practices;* and *virological suppression*) [4, 5, 9]. Following the WHO sampling strategy, we earlier evaluated EWIs on selected ART clinics based on predefined criteria [4–9]. Following the WHO methodology during previous assessments, we found it difficult to identify specific programmatic factors associated with performance of retention in care at 12 months of ART at clinic-, at regional-, or at national-levels [7–9].

We aimed to ascertain the national ART programme performance on retention in care at 12 months of ART and associated factors in Cameroon, for the implementation of an optimal strategy against preventable HIVDR and for a mastering of ARV needs in an era of national treatment uptakes.

## Methods

### Study design and target populations

A cohort-study was conducted from February 23 to 06 March 06, 2015 in all the ten regions of Cameroon, among PLHIV initiating ART in the “*enrolment period*” ranging from October 2013 to December 2013 and followed-up for 12 months (i.e. October 2014 to December 2014). Thus, study “*reporting period*” ranges from October 2013 to November 2014.

### Sampling method

Following a systematic random sampling with unequal probability at two-levels, all ten regions were systematically included in the survey, followed by a random selection of reference treatment centres and HIV management units in each region. Main exclusion criteria were sites with non-significant number of patients (<30) initiating ART within the defined “*enrolment period*”, calculated using the following formula:

$$ \mathrm{n}=\frac{{\mathrm{t}}^2\times \mathrm{p}\left(1 - \mathrm{p}\right)}{{\mathrm{m}}^2} $$

With n = required minimum sample size; t = Z value at a confidence interval of 95% (1.96); p = assumed rate of retention at 12 months of ART; and m = error rate marge at 1% (0.01).

Overall, 56 sites were enrolled and their characteristics described: regional location, geographic settings (urban versus rural), and level of HIV clinic (reference treatment centre versus HIV management unit). In each selected site, patients initiating ART during the “*enrolment period*” were consecutively included until complete sampling for the study (for a total of 5005 in number).

### Data collection

Data were abstracted from ART registers, medical files and pharmacy registers used to monitor patients at the respective ART clinic, and entered into EWI 2 abstraction tool as previously described [9]. Incoherent data were resolved by retrieving additional record documents available at the clinic.

### Quality assurance and data validation

**Fig. 1:**
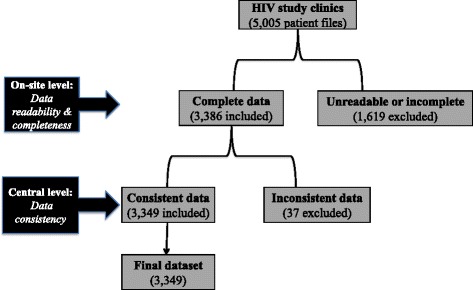
Data collection and validation procedure

To ensure reliability in collected data, only staff (statistician and experienced personnel) trained on HIV data management participated in the data abstraction process. Supervisors were Public Health experts from the central level with field experience on the collection and reporting of data in the ART programme. After on-site validation (mainly for data readability and completeness) on 5005 patient files, abstracted data were centralised at the national level, whereby a second validation (mainly for verifying data consistency) was conducted through a double electronic data-entry; leading to 66.91% inclusion in the final dataset (Fig. 1).

### Data analysis

Interpretation of data was performed according to the definition of EWI 2 as described in Table 1. Chi Square test was used in assessing statistical associations, with *p*-value <0.01 considered significant.

**Table 1:** Definition and data analysis of retention on ART

Title	Definition	Numerator	Denominator	Target
Retention in care at 12 months of ART	Percentage of adults and children known to be alive and on ART 12 months after initiation	Number of adults and children who are still alive and on ART 12 months after initiating treatment	Total number of adults and children (excluding transfers out) who initiated ART and were expected to achieve 12-month outcomes within the reporting period	Desirable or excellent performance: >85%
				Fair performance: 75–85%
				Poor performance: <75%

Legend. *ART* antiretroviral therapy. Were classified as lost to follow-up patients who fail to return for drug pick-up afterthree consecutive months following the last expected pharmacy appointment date

### Availability of data and materials

The data supporting the findings are provided in the main paper and in additional supporting files uploaded as supplement contents with this manuscript.

## Results

### Socio-demographic characteristics of the study population

Of all 3349 PLHIV in the validated dataset from the 56 study sites, distribution according to geographic settings showed that 2491 (74.4%) and 858 (25.6%) were respectively in the urban and rural settings. According to level of HIV clinic, 50.9% were enrolled in a reference treatment centre; while for regional distribution, the Centre, Northwest, Littoral, and Southwest regions were highly represented (17.2, 14.8, 13.8, and 12.3% respectively), as shown in supplemental digital content (Additional file 1: SDC 1).

Out of 3256 PLHIV initiating ART whose gender and age were recorded in the 56 study sites, 2201 (67.6%) were female (gender ratio: two women for one man, *p* < 0.0001); mean age was 36.1 years (+/− 12.2), min-max: 0.16 – 74; and only 4.2% were children or adolescents aged <15 years (Additional file 1: SDC 2).

### Clinical characteristics of the study population

Of the 2163 patients with available clinical classification at ART initiation, 1279 (59.1%) were at the WHO clinical stage III, with 60.0 and 58.7% respectively in the male and female populations.

Of the 2621 patients with available CD4 recorded at ART initiation, a median of 223 CD4 cells/mm^3^ [IQR: 52; 384] was recorded, and the majority (86.5%) had ≤ 350 CD4 cells/mm^3^, while <5% had a CD4 count >500 cells/mm^3^.

All 3349 patients had their initial ART regimens recorded, and predominant regimens were tenofovir (TDF)/3TC/EFV (44%) and zidovudine (AZT)/3TC/NVP (31.6%), as detailed in Table 2.

**Table 2:** Distribution of participants by WHO-staging, CD4 count and initial ART regimens

		Male		Female		Total	
		n	%	n	%	n	%
WHO clinical staging	I	80	11.5%	274	18.6%	354	16.4%
	II	86	12.4%	204	13.9%	290	13.4%
	III	416	60.0%	863	58.7%	**1279**	**59.1%**
	IV	111	16.0%	129	8.8%	240	11.1%
	Total	693	100.0%	1470	100.0%	2163	100.0%
CD4 count	0 to 350	737	89.0%	1541	85.9%	**2278**	**86.9%**
	351 to 500	59	7.1%	164	9.1%	223	8.5%
	>500	32	3.9%	88	4.9%	120	4.6%
	Total	828	100.0%	1793	100.0%	2621	100.0%
Initial ART regiments	AZT/3TC/EFV	142	13.1%	292	12.9%	434	13.0%
	AZT/3TC/NVP	347	32.0%	710	31.3%	**1057**	**31.6%**
	TDF/3TC/EFV	489	45.2%	986	43.5%	**1475**	**44.0%**
	TDF/3TC/NVP	60	5.5%	192	8.5%	252	7.5%
	Others	45	4.2%	86	3.8%	131/	3.9%
	Total	1083	100.0%	2266	100.0%	3349	100.0%

Legend. *ART* antiretroviral therapy, *WHO* World Health Organisation, *3TC* lamivudine, *AZT* zidovudine, *EFV* Efavirenz, *NVP* nevirapine, *TDF* Tenofovir

In bold are the most prevalent data in WHO clinical stage, CD4 count, and initial ART in the study population

### Performance in retention on ART

#### Retention according to geographic settings

Globally, the national level of retention in care 12 months after ART initiation was 60.4% (2023/3349), indicating an overall poor performance in patient retention shortly after ART initiation, with a significantly lower performance in urban (58.5%) compared to rural (66.0%) settings, *p* < 0,0001.

According to regions, retention capacity varied considerably, with the highest performance reported in the Littoral Region (75.1%), followed by the East, Centre and Adamawa regions (70.2, 64.8, and 63.4% respectively). Thus, no region in the country had an excellent/desirable performance, and only one region (Littoral) had a fair performance, while the West and Far-North regions that registered the poorest performances (42.8 and 47.4% respectively), as shown in Table 3.

**Table 3:** Retention on ART by geographic settings and by region

Regions	Urban		Rural		Total	
	n/N	%	n/N	%	n/N	%
Adamawa	130/205	63.4%	NA^a^	NA^a^	130/205	63.4%
Centre	109/162	67.3%	265/415	63.8%	374/577	64.8%
East	91/155	58.7%	88/100	88.0%	179/255	70.2%
Far-North	66/163	40.5%	26/31	83.9%	92/194	47.4%
Littoral	346/461	75.1%	NA^a^	NA^a^	346/461	**75.1%**
North	104/166	62.7%	35/69	50.7%	139/235	59.1%
Northwest	141/264	53.4%	152/233	65.2%	293/497	59.0%
West	133/311	42.8%	NA^a^	NA^a^	133/311	42.8%
South	105/201	52.2%	NA^a^	NA^a^	105/201	52.2%
Southwest	232/413	56.2%	NA^a^	NA^a^	232/413	56.2%
Total	1457/2501	58.3%	566/848	66.7%	2023/3349	60.4%

Legend. n: frequency; %: proportion of patients in care for each region and in the total study population

^a^: NA (Not applicable), due to selection of no rural site following the random sampling

In bold are the most prevalent data on retention from the national regions

#### Retention according to HIV clinic levels

According to clinic levels, reference treatment centres had an overall performance of 54.2% against 66.8% in HIV management units, indicating paradoxically a better capacity of low-level clinics in retaining patients on ART. Of note, the HIV management unit with the highest performance had 93.1% (in the East region) while the reference treatment centre with the highest performance had 83.5% (in the Littoral region), indicating respectively an excellent and a fair capacity for retention on ART (Additional file 1: SDC 3).

### Factors associated with levels of retention on ART after 12 months

#### Association between demographic profile and retention on ART

The overall gender distribution at 12 months after ART initiation revealed that 62.0% (1405) women compared to 57.1% (618) men were still in care, indicating a significantly better adherence of women to the national ART program, *p* = 0.007. Similar trends were observed when comparing retention in care between women (68.3%) and men (63.6%) followed-up in HIV management units (*p* = 0.057), as well as in reference treatment centres (55.8% versus 50.9% respectively), *p* = 0.018.

According to age groups, paediatrics (children and adolescents < 15 years) had similar retention rates compared to older patients (58.8 and 60.3%, respectively, *p* = 0.730); with a slightly lower performance observed among children aged 5–9 years (47.2% versus 63% for other children age, *p* = 0.099), as shown in Table 4.

**Table 4:** Retention on ART 27 by gender, age and level of HIV clinics

		Reference treatment centre		HIV management unit		Total	
		n/N	%	n/N	%	n/N	%
Gender	Male	284/558	50.9%	334/525	63.6%	618/1081	57.2%
	Female	641/1148	55.8%	764/1118	68.3%	1405/2266	**62.0%**
Age range	Less than 4 years	13/25	52.0%	28/40	70.0%	41/65	**63.1%**
	5*–*9 years	8/19	42.1%	9/17	52.9%	17/36	47.2%
	10*–*14 years	7/17	41.2%	15/18	83.3%	22/35	62.9%
	15*–*19 years	12/20	60.0%	15/30	50.0%	27/50	54.0%
	20*–*24 years	66/127	52.0%	74/108	68.5%	140/235	59.6%
	25*–*29 years	134/260	51.5%	168/233	72.1%	302/493	61.3%
	30*–*34 years	169/312	54.2%	182/294	61.9%	351/606	57.9%
	35*–*39 years	134/240	55.8%	187/280	66.8%	321/520	61.7%
	40*–*44 years	126/234	53.8%	158/230	68.7%	284/464	61.2%
	45*–*49 years	84/157	53.5%	97/149	65.1%	181/306	59.2%
	50*–*54 years	71/119	59.7%	64/102	62.7%	135/221	61.1%
	55 years and	53/107	49.5%	88/118	74.6%	141/225	62.7%
Age range	Children	28/61	45.9%	52/75	69.3%	69.3%	58.8%
	Adults	849/1575	53.9%	1033/1544	66.9%	1882/3119	**60.3%**
Total	Total	925/1706	54.2%	1098/1643	66.8%	2023/3349	60.4%

Legend. n: frequency; %: proportion of patients in care for each region and in the total study population; Reference treatment centre provides mentorship to HIV management units within his health area. In bold are the most prevalent data on retention on ART according to gender and to age range

#### Association between clinical characteristics and retention on ART

Clinical analysis revealed that 12 months retention on ART seems decreasing from WHO clinical stage I to IV, with the highest performance (63.3%) reported among patients with at WHO clinical stage I while the poorest (55.4%) was among ones at WHO clinical stage IV, *p* = 0.007. With a median of 223 CD4 cells/mm^3^, we observed a slightly higher retention on ART at 12 months for patients who started treatment with 351–500 cells/mm^3^ (65.9%) compared to other categories (59.86% for ones with = <350 CD4 cells/mm^3^ and with >500 CD4 cells/mm^3^, *p* = 0.077). Overall, clinical parameters showed low rates of retention (below performance limit of 75%), thus indicating higher risk of HIVDR emergence (Table 5). However, retention performance at all clinical parameters was significantly greater in HIV management units compared to reference treatment centres (66.8% versus 54.2%, respectively); *p* < 0.0001.

**Table 5:** Retention on ART according to clinical parameters

		Reference treatment centres		HIV management units		Total	
		n/N	%	n/N	%	n/N	%
WHO clinical stage	I	100/183	54.6%	124/171	72.5%	224/354	**63.3%**
	II	71/158	44.9%	94/132	71.2%	165/290	56.9%
	III	299/545	54.9%	493/734	67.2%	792/1279	61.9%
	IV	33/99	33.3%	100/141	70.9%	133/240	55.4%
CD4 count	0–350	576/1114	51.7%	787/1164	67.6%	1363/2279	59.8%
	351–500	55/93	59.1%	92/130	70.8%	147/223	**65.9%**
	Above 500	19/35	54.3%	54/85	63.5%	73/120	60.8%
Total		925/1706	54.2%	1098/1643	66.8%	2023/3349	60.4%

Legend. n: frequency; %: proportion of patients in care for each region and in the total study population; Reference treatment centre provides mentorship to HIV management units within his health area. In bold are the most prevalent data on retention according to WHO clinical stage and CD4 count

### Classification levels of retention on ART by health facilities

Overall, only six out of 56 (10.71%) health facilities had acceptable performance for retention on ART at 12 months, among which 5.36% (3/56) with excellent (>85%) performance (Ngaoundéré Presbyterian Hospital [93.1%], CBC of Mboppi [89.5%], PR Garoua Boulai [88%]), and 5.36% (3/56) with fair (75–85%) performance (HD Pette [83.9%], Douala Laquintinie Hospital [83.5%], and DH Mamfe [81.3%]), which were mostly HIV management units from diverse regions in Cameroon. Poorest performances were observed from an HIV management unit (NKAMBE DH [6.2%]) and a reference treatment centre (Yaoundé General Hospital [18.9%]). Thus, 89.29% of health facilities had a poor (<75%) performance in retention on ART at 12 months; indicating an overall high risk of HIVDR emergence in these ART clinics.

## Discussion

As an indicator of potential loss to follow-up in ART programs and a tool for estimates in ARV procurement and supply, patient retention in care also serves in tailoring adherence support towards a better control of preventable HIVDR [5, 6]. As compared to previous studies in Cameroon [7–9], our findings provide a prime estimate of the country capacity both in enrolling and in retaining patients on care 12 months after ART initiation. Importantly, the random sampling, across 56 ART clinics from all 10 regions of Cameroon, is innovative as it generates more meaningful evidence-based interventions, compared to the WHO primary sampling strategy [5, 6].

From this random sampling, only 60% of patients were still in care at 12 months after ART initiation, resulting in a poor national performance (i.e. <75%) for patient retention on ART, translated into 40% potential lost to follow-up [12]. As loss to follow-up are known as possible HIVDR, national ART programs in SA has ~40% likelihood of experiencing HIVDR emergence in a short-medium run [10, 11]. Of note, only 10.71% ART clinics and only one region (Littoral) had acceptable performances (excellent or fair), suggesting that ~90% of ART clinics as well as 90% of regions are experiencing high risks of HIVDR due to poor retention on ART, similar to findings from other SSA-settings [13]. The high performance observed in a reference centre of the Littoral is likely due to ongoing project that provides additional support for patient monitoring onsite, thus fostering adherence to care, as earlier addressed [9]. Compared to recently conducted EWI survey, performance was lower (10.71% versus 76.92%), and worse in urban settings [9]; a disparity likely attributed to the random sampling that gives broaden and in-depth appraisals of program functioning at all levels. Retention was also significantly lower (*p* < 0.0001) in HIV reference treatment centres than in management units. This outcome could be partly attributed to the burnout syndrome (heavy workload) which appears higher in reference treatment centres as we earlier reported within this national context (74.4% patients versus 25.6% in management units), calculated per site based on the number of trained physicians, nurses, pharmacists/clerks, biologists/laboratory technicians, counsellors, data managers, community relay agents involved on the routine management of PLHIV [8]. Advanced implementation of task shifting and decentralisation might help alleviating the burnout syndrome [8, 13–15]. Retention at 12 months of ART was also problematic in African countries and beyond, supporting the needs to explore other local factors (geographical landscape around a single ART clinic, availability of transportation facilities, effectiveness of decentralisation, etc.) that may be warrant public health actions [12–19].

Our study delineates additional reasons underscoring this poor national retention on ART. Of note, poor retention was more concerning in men (*p* = 0.007), thus requiring intensified adherence support to men on ART following their daily realities and challenges [18]. As previously reported, African women are generally known to be more adherent to ART programs [20, 21]; this might also be attributed to attention offered to women through the PMTCT program and cascade of health care for mother and child (an information not captured in our dataset), and the eventual non-adherence of male patients [22]. Due to the limited representativeness of children (<15 years), retention on ART according to age merits further investigations, with linkage to virological suppression according to age groups [21, 23].

The significant risk (*p* = 0.007) of poor retention on ART among potential symptomatic patients (i.e. 55.6% WHO clinical stages I/II/III:) versus potential healthy/asymptomatic patients (i.e. 59.1% WHO clinical stage I) indicates the need to implement an active monitoring/tracking system for patients suffering of any disease at ART initiation, as well as social and financial barriers [21, 23]. This underscores that early ART initiation would promote retention on ART [21]. Moreover, median CD4 (223 cells/mm^3^) indicates an overall delay initiation of ART (though better than previous reports: 123 and 163 CD4 cells/mm^3^) [12, 24], which in turns predict poor outcomes both in retention and in clinical benefits [21, 25]. Despite no significance between CD4 range and retention levels (*p* = 0.077), severely immune compromised patients would likely default from care due to clinical manifestations [23, 26], thus implying policies for timely ART initiation as we transition to ≤500 CD4 cells/mm^3^ for initiation [3, 4, 19].

Decreasing retention was observed with periods of ART initiation (October-December, p <0.02), suggesting further understanding of related temporal barriers on defaulters or lost to follow-up [24], especially in the frame of the current “*90-90-90*” goals in RLS [27]. Thus, innovative approaches (community-based test-and-treat, mobile clinics, use of SMS for recalls, adherence clubs, and point-of-care monitoring) are highly necessary to curve the epidemic, especially within SSA settings [27–33].

### Study strengths

Including 56 ART clinics, in all the ten national regions, gives room for greater country representativeness (compared to previous studies [7–9]), further strengthened by random sampling at regional level. Findings would directly contribute for planning, monitoring and evaluation of national ART program, and for policy-implementation in other RLS with similar challenges [5, 6].

### Study limitations

Data rejection (~33%) weakens our expected study strengths, thus indicating needs for continuous training on data reporting in the continuum of ART programs within such RLS. Rural settings were limited, suggesting a two-level systematic sampling, followed by randomisation only within unique geographical strata (urban vs. rural; reference centre vs. management units, etc.) [33]. Study-period (1-year) was a limited to assess retention rates according to different ART-regimens, thus requiring long-term cohort-studies while transitioning towards new ART recommendations [4]. Predictors of delayed ART initiation, and other aspects of adherence to ART (on-time drug pickup, pill count, self-reported adherence, and/or drug dosage) should be covered subsequently, for optimal policies.

### Context-specific programmatic lessons

Despite the high capacity of the national AIDS program in enrolling patients on ART, poor performance in retaining these patients in care is a serious treat for ART effectiveness and coverage. Interestingly, results predict a better life expectancy for women over men, who appear less likely than women to seek and adhere to ART [22]. On a separate note, active search for patients out of care is mandatory, including community engagement, to sustain ART cohorts and routine drug procurement. Accelerating task shifting and decentralisation would alleviate the tentative heavy workload. Therefore, in routine practice, follow-up/adherence support should mainly target male and symptomatic patients initiating ART nationwide. Programmatic assessments may integrate quality of care indicators [34], affordable monitoring assays [35–37], and simplified/cost-effective ARV drug provision strategies [38, 39] for the national ART performance, in a context of increasing risks of HIVDR [40–42].

## Conclusions

One year after ART initiation in Cameroonian clinical settings, retention in care remains below acceptable standards, suggesting risks of HIVDR emergence nationwide. Adherence interventions should focus primarily on male and symptomatic patients, especially within reference ART clinics. Such systematic-random sampling strategy should be further strengthened, in the frame of the global perspectives for HIVDR prevention in RLS.

## Additional file

Additional file 1:
**SDC 1.** Distribution of the study population by geographic settings, by level of HIV clinic, and by region. Geographical setting is reported as rural or urban; level of HIV clinic is reported as reference treatment centres or HIV management units; regions represent all ten geographical regions of Cameroon. All data are reported for male, female and the overall population. **SDC 2.** Distribution of study population by gender and by age. Data are represented in age range of five years; an aggregated data for adults versus children is also provided. All data are reported for male, female and the overall population. **SDC 3.** Retention on ART by level of HIV clinic. Retention in care is provided for reference treatment centres and for HIV management units, reported for each region and for the overall geographical regions of Cameroon. (DOCX 111 kb)

## Abbreviations

3TC: Lamivudine
ART: Antiretroviral therapy
ARV: Antiretroviral
AZT: Zidovudine
EFV: Efavirenz
EWI: Early warning indicators
FTC: Emtricitabine
HIV: Human immunodeficiency virus
HIVDR: HIV drug resistance
NVP: Nevirapine
PLHIV: People living with HIV
PMTCT: Prevention of mother-to-child transmission
TDF: Tenofovir
WHO: World Health Organization
