# Supplementary material for: Predictors of poor retention on antiretroviral therapy as a major HIV drug resistance early warning indicator in Cameroon: results from a nationwide systematic random sampling
Source: BMC Infect Dis. 2016 Nov 15;16:678. doi: 10.1186/s12879-016-1991-3 (PMC5111226; doi:10.1186/s12879-016-1991-3)
Supplement: Additional file 1: — SDC 1. Distribution of the study population by geographic settings, by level of HIV clinic, and by region. Geographical setting is reported as rural or urban; level of HIV clinic is reported as reference treatment centres or HIV management units; regions represent all ten geographical regions of Cameroon. All data are reported for male, female and the overall population. SDC 2. Distribution of study population by gender and by age. Data are represented in age range of five years; an aggregated data for adults versus children is also provided. All data are reported for male, female and the overall population. SDC 3. Retention on ART by level of HIV clinic. Retention in care is provided for reference treatment centres and for HIV management units, reported for each region and for the overall geographical regions of Cameroon. (DOCX 111 kb) [file 12879_2016_1991_MOESM1_ESM.docx]

**Supplemental Digital Contents (SDC)**

**SDC 1: Distribution of the study population by geographic settings, by level of HIV clinic, and by region.**

|  | | Male | | Female | | Total | |
| --- | --- | --- | --- | --- | --- | --- | --- |
|  |  | ***n*** | ***%*** | ***n*** | ***%*** | ***N*** | ***%*** |
| Geographic setting | Urban | 801 | 74.0% | 1690 | 74.6% | 2491 | 74.4% |
|  | Rural | 282 | 26.0% | 576 | 25.4% | 858 | 25.6% |
|  | Total | 1083 | 100.0% | 2266 | 100.0% | 3349 | 100.0% |
| Level of HIV clinic | Reference treatment centres | 558 | 51.5% | 1148 | 50.7% | 1706 | 50.9% |
|  | HIV management units | 525 | 48.5% | 1118 | 49.3% | 1643 | 49.1% |
|  | Total | 1083 | 100.0% | 2266 | 100.0% | 3349 | 100.0% |
| Region | Adamawa | 65 | 6.0% | 140 | 6.2% | 205 | 6.1% |
|  | Centre | 181 | 16.7% | 396 | 17.5% | 577 | 17.2% |
|  | East | 77 | 7.1% | 178 | 7.9% | 255 | 7.6% |
|  | Far-North | 73 | 6.7% | 121 | 5.3% | 194 | 5.8% |
|  | Littoral | 130 | 12.0% | 331 | 14.6% | 461 | 13.8% |
|  | North | 68 | 6.3% | 167 | 7.4% | 235 | 7.0% |
|  | Northwest | 172 | 15.9% | 325 | 14.3% | 497 | 14.8% |
|  | West | 113 | 10.4% | 198 | 8.7% | 311 | 9.3% |
|  | South | 61 | 5.6% | 140 | 6.2% | 201 | 6.0% |
|  | Southwest | 143 | 13.2% | 270 | 11.9% | 413 | 12.3% |
|  | Total | 1083 | 100.0% | 2266 | 100.0% | 3349 | 100.0% |

**SDC 2: Distribution of study population by gender and by age.**

|  | | Male | | Female | | Total | |
| --- | --- | --- | --- | --- | --- | --- | --- |
|  | | ***n*** | ***%*** | ***n*** | ***%*** | ***n*** | ***%*** |
| Age range | Less than 5 years | 27 | 2.6% | 38 | 1.7% | 65 | 2.0% |
|  | 5 - 9 years | 16 | 1.5% | 20 | 0.9% | 36 | 1.1% |
|  | 10 - 14 years | 16 | 1.5% | 19 | 0.9% | 35 | 1.1% |
|  | 15 - 19 years | 12 | 1.1% | 38 | 1.7% | 50 | 1.5% |
|  | 20 - 24 years | 27 | 2.6% | 208 | 9.5% | 235 | 7.2% |
|  | 25 - 29 years | 95 | 9.0% | 398 | 18.1% | 493 | 15.1% |
|  | 30 - 34 years | 175 | 16.6% | 431 | 19.6% | 606 | 18.6% |
|  | 35 - 39 years | 190 | 18.0% | 330 | 15.0% | 520 | 16.0% |
|  | 40 - 44 years | 198 | 18.8% | 266 | 12.1% | 464 | 14.3% |
|  | 45 - 49 years | 132 | 12.5% | 174 | 7.9% | 306 | 9.4% |
|  | 50 - 54 ans | 85 | 8.1% | 136 | 6.2% | 221 | 6.8% |
|  | 55 years and above | 82 | 7.8% | 143 | 6.5% | 225 | 6.9% |
|  | Total | 1055 | 100.0% | 2201 | 100.0% | 3256 | 100.0% |
| Age range | Children | 59 | 5.6% | 77 | 3.5% | 136 | 4.2% |
|  | Adults | 996 | 94.4% | 2124 | 96.5% | 3120 | 95.8% |
|  | Total | 1055 | 100.0% | 2201 | 100.0% | 3256 | 100.0% |

**SDC 3: Retention on ART by level of HIV clinic.**

| Region | Reference treatment centres | | HIV management units | | Total | |
| --- | --- | --- | --- | --- | --- | --- |
|  | ***n*** | ***%*** | ***n*** | ***%*** | ***n*** | ***%*** |
| Adamawa | 76 | 51.7% | 54 | 93.1% | 130 | 63.4% |
| Centre | 215 | 64.8% | 159 | 64.9% | 374 | 64.8% |
| East | 91 | 58.7% | 88 | 88.0% | 179 | 70.2% |
| Far-North | 92 | 47.4% | **0** | **0.0%** | 92 | 47.4% |
| Littoral | 91 | 83.5% | 255 | 72.4% | 346 | 75.1% |
| North | 104 | 62.7% | 35 | 50.7% | 139 | 59.1% |
| Northwest | 81 | 46.8% | 212 | 65.4% | 293 | 59.0% |
| West | 68 | 36.8% | 65 | 51.6% | 133 | 42.8% |
| South | 51 | 42.9% | 54 | 65.9% | 105 | 52.2% |
| Southwest | 56 | 44.4% | 176 | 61.3% | 232 | 56.2% |
| Total | **925** | **54.2%** | **1098** | **66.8%** | **2023** | **60.4%** |
